# Surveillance of tick-borne encephalitis in emerging risk areas in southern Sweden: a retrospective case finding study

**DOI:** 10.1007/s10096-022-04509-1

**Published:** 2022-11-02

**Authors:** Mattias Waldeck, Niclas Winqvist, Gunnel Henriksson, Robert Dyrdak, Bo Settergren, Per-Eric Lindgren

**Affiliations:** 1 Regional Office of Communicable Disease Control and Prevention, Malmö, Region Skåne Sweden; 2 Inflammation and Infection, Department of Biomedical and Clinical Sciences, Linköping University, Linköping, Sweden; 3 Department of Infectious Diseases, Central Hospital Kristianstad, Kristianstad, Sweden; 4 Clinical Infection Medicine, Department of Translational Medicine, Lund University, Lund, Sweden; 5 Department of Clinical Microbiology, Skåne University Hospital, Lund, Sweden; 6 Department of Laboratory Medicine, Division of Medical Microbiology, Lund University, Lund, Sweden; 7 Department of Clinical Microbiology, Karolinska University Hospital, Stockholm, Sweden; 8 Department of Microbiology, Tumor and Cell Biology, Karolinska Institutet, Stockholm, Sweden; 9 Clinical Microbiology, Laboratory Medicine, County Hospital Ryhov, Jönköping, Sweden

**Keywords:** TBEV, Encephalitis, Meningitis, Tick-borne infection, Epidemiological monitoring

## Abstract

Tick-borne encephalitis (TBE) is an emerging infection causing CNS infection of various severity. Good knowledge of the incidence in the population and defined risk areas is important in risk communication and vaccination recommendations. The aim of this study was to investigate potential underreporting by retrospectively diagnose TBE among patients with viral CNS infections of unknown etiology in a region with emerging risk areas for TBE, and define variables associated with performed TBE serology at the time of infection. Epidemiological data and microbiological diagnostics of cases with viral CNS infection of unknown etiology treated at departments of infectious diseases and pediatrics in Skåne County during 2000–2012 were investigated. Analyses to evaluate variables associated with performed TBE serology at the time of infection were performed. Retrospective TBE serology was performed on stored blood samples when available. TBE serology was already performed at the time of CNS infection in 193 out of 761 cases. Department, type of clinical manifestation, time period of illness, and whether Borrelia serology had been performed were independent variables associated with having had TBE serology performed or not at the time of illness. Only one of 137 cases, where samples could be retrospectively analyzed for TBE, turned out positive. This study shows a low frequency of TBE sampling among patients with meningoencephalitis in a region with emerging risk for TBE. A higher awareness of TBE as differential diagnosis could contribute to earlier detection of new risk areas and adequate preventive advice to the public.

## Introduction

Viral infections affecting the central nervous system (CNS) present as meningitis, meningoencephalitis, encephalitis, or meningoencephalomyelitis with different degrees of severity, ranging from mild meningitis to encephalitis with disabling sequalae and even death. Several different viruses are known to cause CNS infections, and different viruses have different epidemiology [1]. However, only in up to 70% of individuals diagnosed with viral meningitis and in about 50% diagnosed with encephalitis, an etiologic agent is found [2–5]. In Sweden, viral CNS infections are, according to the Swedish Communicable Disease Act, notifiable to the regional office for disease control and prevention. Notification is mandatory for the microbiological laboratory identifying the virus, as well as for the clinicians treating the patients. Although an etiologic agent cannot always be expected to be found, almost all the notifications concern patients where an etiologic agent has been identified. Hence, a considerable number of viral CNS infections are never reported and thereby not surveyed.

**Fig. 1:**
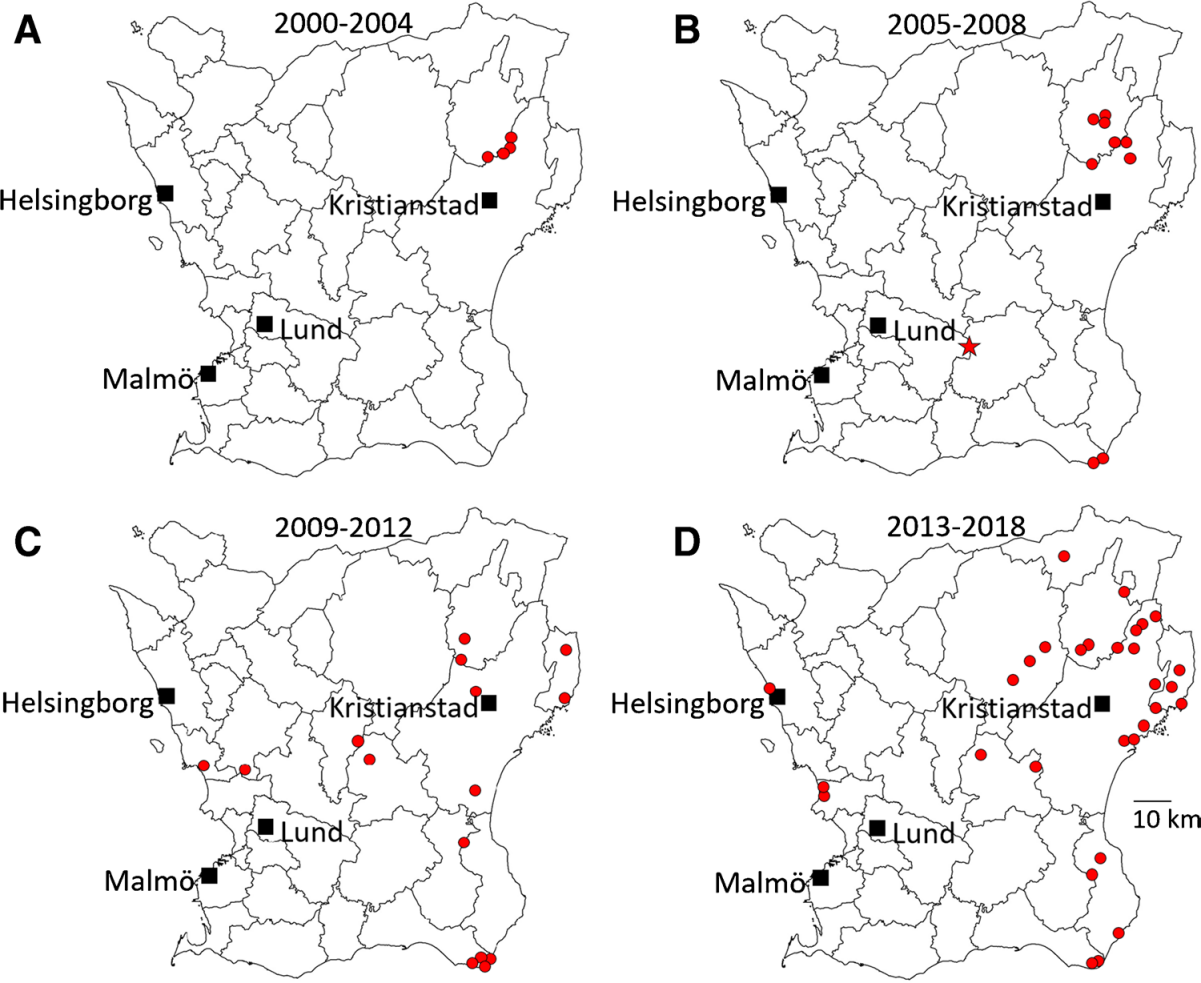
Map of Skåne County and place of infection for TBE cases (red dots) diagnosed in the routine health care during the time periods of the study, **A**–**C**, and the cases the following years after the study period, **D**. The red star in map **B** marks the probable geographical site of infection for the TBE case retrospectively diagnosed in this study

Tick-borne encephalitis (TBE) was first described in Sweden in the 1950s in Stockholm County and was for decades mostly restricted to that region [6, 7]. TBE virus (TBEV) is in Sweden spread by the common tick, *Ixodes ricinus*, but the prevalence of ticks harboring TBEV is low, even in TBE endemic areas [8]. During the last decades, there has been an increasing number of human TBE cases, and dissemination into new focus areas in Sweden as well as in other European countries [9–11]. The first TBE focus in Skåne County, the southernmost part of Sweden, was identified in 2002 in a restricted area of the northeastern part of the county [12, 13]. Since then, the annual number of TBE cases infected in Skåne County has gradually increased as well as the number and expansion of risk areas (Fig. 1).

Compared to other regions in Sweden, Skåne County still has a very low incidence of TBE: 0.8–1.4/100,000 inhabitants (2018–2021) [14]. However, recognition and laboratory confirmation of all TBE cases are essential for accurate assessment of risk areas for TBE, since preventive measures are available, especially vaccination of people at risk. TBE is usually easy to diagnose with detection of IgM and IgG antibodies against TBEV in blood samples (TBE serology). Furthermore, alertness of diagnosing emerging infections is crucial in the context of the overall changed panorama of vector-borne infections due to the ongoing climate changes.

The aim of this study was to investigate to what extent TBE cases have been underdiagnosed during the period 2000–2012 in Skåne County, a period when TBE emerged in southernmost Sweden. We also investigated to what extent of the patients with viral CNS infections of unknown etiology TBE analysis had been performed, and which factors were associated with performing TBE serology, at the time of illness.

## Methods

### Study design and setting

We performed a descriptive, retrospective cross-sectional study of patients diagnosed with presumed viral CNS infection at the departments of infectious diseases and pediatrics, respectively, in Skåne County during 2000–2012. Skåne County had a population of about 1.2 million inhabitants towards the end of the study period (2012). The hospitals in the four largest cities, Malmö, Lund, Helsingborg, and Kristianstad, all have their own departments of infectious diseases and pediatrics whereas the other five smaller hospitals in the county do not. Departments of infectious diseases treat adult patients and departments of pediatrics, children up to 18 years old, although some overlapping occurs. Analysis of TBE serology has been done at the Skåne University Hospital in Lund since 2007 and prior to that, samples for TBE serology were sent to the Microbiological Laboratory at The Public Health Agency of Sweden, Solna, or to the Clinical Microbiological Laboratory at Karolinska University Hospital, Solna, Sweden. *Borrelia* serology has been performed at different laboratories in Skåne Region during the study period.

### Study population, data sources, and data collection

For every consultation at an outpatient ward or when treated as an inpatient, one or more diagnosis codes according to the International Statistical Classification of Diseases and Related Health Problems 10th Revision (ICD-10 codes) are given to classify the disease or symptoms of the patient, and the codes are then registered in the health care registry. ICD-10 codes relevant for the study population were identified, and all that comprised viral or unspecified infection of the CNS with unspecified etiology were included (Table 1). Patients treated as inpatients or outpatients at the four departments of infectious diseases and the four departments of pediatrics in Skåne County, with the specified ICD-10 codes during 2000–2012, formed the study population. Data retrieved from the health care registry included gender, personal identification number, date of admission and discharge or, for outpatients, date for consultation, and ICD-10 codes given. Children under 1 year of age were excluded due to the improbability to contract TBE as an infant. Health care registry data were available from year 2000 in Kristianstad (for outpatients at the department of infectious diseases from 2004) and Helsingborg, and from 2001 in Lund and Malmö. For inpatients, health care registry data were available from year 2000 at the departments of infectious diseases and pediatrics in Kristianstad and Helsingborg, and from 2001 at the departments of infectious diseases and pediatrics in Lund and Malmö. For outpatients, data were available from year 2000 at the departments of infectious diseases in Helsingborg and pediatrics in Helsingborg and Kristianstad, from 2001 at the departments of infectious diseases and pediatrics in Lund and Malmö, and from 2004 at the department of infectious diseases in Kristianstad. In the statistical analysis, the ICD-10 codes were grouped into meningitis (A87.9, G03.9), encephalitis (A83.9, A84.9, A85.2, A86.9, G04.9), or unspecified CNS infection (A81.9, A89.9).

**Table 1:** List of ICD-10 codes comprising viral CNS infections of unknown etiology included in the study

ICD-10 code	Diagnose
A81.9	Atypical viral infection in the CNS, unspecified
A83.9	Viral encephalitis transmitted by mosquitoes, unspecified
A84.9	Viral encephalitis transmitted by ticks, unspecified
A85.2	Viral encephalitis transmitted by arthropods, unspecified
A86.9	Viral encephalitis, unspecified
A87.9	Viral meningitis, unspecified
A89.9	Viral infection in the CNS, unspecified
G03.9	Meningitis, unspecified
G04.9	Encephalitis, myelitis, and encephalomyelitis, unspecified

For each patient with any of the specified ICD-10 codes, the microbiological test results at the time of the illness were explored by access to the database of the Clinical Microbiological Laboratory at the Skåne University Hospital. For patients with no information regarding TBE serology, an inquiry to the Microbiological Laboratory at The Public Health Agency of Sweden and the Clinical Microbiological Laboratory at Karolinska University Hospital, Solna, was sent to explore if TBE serology had been performed at these laboratories. If an etiological agent already was found, the patient was excluded from further analysis. For each patient, information whether a TBE serology had been performed or not, as well as *Borrelia* serology at the time of the CNS infection, was recorded. Data regarding gender, age at the time of infection, year and month of the infection, treating department, and ICD-10 code given were also registered.

### Retrospective TBE serology

For patients where TBE serology had not been performed at the time of illness, and where a stored frozen blood sample from the time of illness was available in the biobank, TBE serology was performed retrospectively after informed consent was obtained from the patients or, for children, their guardians. The patients/guardians were asked for informed consent via mail or, in some cases, by phone. Patients that had their CNS infection during January and February were excluded from retrospective TBE analysis because of the improbability of contracting a tick-borne infection in Skåne County during that period of the year. TBE serology was performed at the Clinical Microbiology Laboratory at Skåne University Hospital in Lund. A commercial enzyme-linked immunosorbent assay (ELISA) from Euroimmun® for detection of IgM and IgG antibodies against TBEV (anti-TBE virus ELISA IgM/anti-TBE virus ELISA IgG, Euroimmun, Lübeck, Germany), used in the routine diagnosis of TBE, was used [15]. Case definitions according to The Public Health Agency of Sweden were used [16]. Detection of both IgM and IgG antibodies against TBEV was assessed as ongoing TBE infection at the time of the illness. Detection of only IgM antibodies against TBEV was considered a possible ongoing TBE infection, whereas detection of only IgG antibodies against TBE was considered a previous infection or result of immunization against TBE. Any positive result was followed up with telephone interviews with the patients to find out probable place for contracting the TBE infection.

### Surveillance of TBE

TBE has been a notifiable disease in Sweden since 1st of July 2004. However, before that, only one laboratory in Sweden performed TBE serology, and hence, there are robust data on TBE cases in Sweden also prior to 2004. Surveillance data on TBE was retrieved from the database of the Regional Office for Communicable Disease Control and Prevention in Skåne County. Information about place of infection was retrieved from interviews with the patients in the routine surveillance of TBE at the Regional Office for Communicable Disease Control and Prevention. For those cases assessed to have contracted the disease in Skåne during the study period, the geographical site of infection for each case was plotted on a map in one of three time periods of the study. Cases in the period after the study were plotted on a fourth map (Fig. 1).

### Statistical methods

The *χ*^2^-test was used in a univariate analysis to assess variables associated with cases where TBE serology was already performed at the time of infection (dependent variable). In case any individual cell had less than five counts, the Fisher’s exact test replaced the *χ*^2^-test. A binary logistic regression analysis was subsequently used in a multivariate model to assess independent variables associated with performed TBE serology. Starting with variables with the strongest associations, variables with a *p*-value < 0.20 in univariate analysis were entered one by one. Using *p* < 0.05 as a threshold for likelihood ratio test at each step, variables remained if fitness of the model was improved. In addition to city, gender, type of infection, *Borrelia* serology performed, and time period of infection, included in the univariate analysis, month of illness was added in the multivariate model. Data from the departments of infectious diseases and pediatrics were analyzed separately but the same outcome variables were used in the multivariate analysis for both type of departments, even if variables in some univariate analysis did not reach *p*-value < 0.20. Odds ratios and 95% confidence intervals were calculated. In the sub-population of patients where *Borrelia* serology had been analyzed at the time of infection, indicating an assessment of possible tick exposure at the time of infection, a separate multivariate analysis with the same outcome variables was performed. All analyses were performed using IBM SPSS® Statistics Version 25.

### Ethical considerations

The study was conducted with the approval of the Regional Ethical Review Board at Lund University, Sweden (DNR: 2014/246). Individuals eligible for retrospective TBE analysis were contacted by mail and were requested to return a written informed consent allowing us to use stored blood samples for subsequent analyses. For children, up to 18 years old, informed consent was given by their guardians.

## Results

**Fig. 2:**
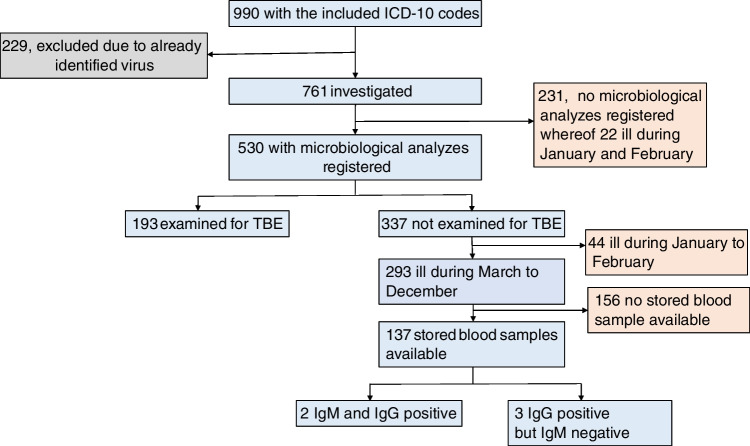
Flow chart of the study. The numbers refer to cases

In total, 990 disease incidents (onward referred to as cases), rendering one of the specified ICD-10 codes, occurred at the departments of infectious diseases or pediatrics during the study period. Despite the ICD-10 code, 229 (23%) were excluded because a viral etiology was already identified and registered in the database of the microbiological laboratory (Fig. 2). The characteristics of the remaining 761 cases are summarized in Table 2. Among the 761 cases, 31 of them were due to recurrent disease incidents in 14 individuals during the study period.

**Table 2:** Characteristics of the 761 included cases

Characteristics	Department of infectious diseases		Department of pediatrics	
	Number of cases	Percent	Number of cases	Percent
Total	587		174	
Gender				
Female	305	52	70	40
Male	282	48	104	60
Age [median (IQR), years]	35 (28–49)		9 (5–13)	
Female	35 (27–46)		10 (4–14)	
Male	36 (29–52)		8 (5–12)	
City				
Helsingborg	79	13	43	25
Kristianstad	99	17	35	20
Lund	239	41	55	32
Malmö	170	29	41	24
ICD-10 code				
A81.9	1	0.2	0	
A83.9	0		0	
A84.9	1	0.2	0	
A85.2	0		0	
A86.9	75	13	31	18
A87.9	439	75	71	41
A89.9	3	0.5	1	0.6
G03.9	15	3	14	8
G04.9	53	9	57	33
Type of infection (%)				
Meningitis	454	77	85	49
Female	261	58	35	41
Male	193	42	50	59
Encephalitis	129	22	88	51
Female	40	31	34	39
Male	89	69	54	61
Unspecified CNS infection	4	0.7	1	0.6
Female	4		1	
Male	0		0	
Samples for retrospective TBE analysis available				
Helsingborg	16		13	
Kristianstad	12		5	
Lund	32		10	
Malmö	40		9	

Meningitis was a more common clinical manifestation compared to encephalitis among adults (77% vs. 22%), whereas meningitis and encephalitis among children were more evenly distributed. The overall gender distribution was equal among adults, but among children, there was a predominance (60%) of boys. However, an uneven gender distribution was observed in the different types of CNS infections, with encephalitis being more frequent among men (69%) and meningitis more frequent among adult women (58%) (Table 2). Among children, boys dominated in both meningitis and encephalitis. TBE serology was performed at the time of infection in 25% (193/761) of all cases and was significantly more common at departments of infectious diseases compared to departments of pediatrics (28% vs.16%; odds ratio (OR): 2.0; 95% confidence interval (CI): 1.3–3.2; *p* < 0.001). At the departments of infectious diseases, cases of encephalitis were significantly more likely to have had a TBE serology performed at the time of infection compared to cases of meningitis (56% vs. 20%; OR: 5.0; 95% CI: 3.2–7.6; *p* < 0.001; Table 3). This difference was not observed at departments of pediatrics (*p* = 0.34). Data on whether *Borrelia* serology was performed at the time of infection was missing for 225 (38%) and 80 (46%) cases treated at departments of infectious diseases and pediatrics, respectively. The missing data on *Borrelia* serology was due to difficulties in retrieving these data from different laboratories that performed it during the study period.

**Table 3:** Univariate and multivariate analysis with performed TBE serology as dependent variable

	Total	TBE serology performed		TBE serology not performed		Univariate analysis			Multivariate analysis		
		*n*	%	*n*	%	OR	95% CI	*p*-value	OR	95% CI	*p*-value
Department of infectious diseases											
City											
Helsingborg	79	15	19	64	81	Ref					
Kristianstad	99	53	54	46	46	4.9	2.4–9.8	** < 0.001**	51.6	14.3–186	** < 0.001**
Lund	239	54	23	185	77	1.2	0.6–2.4	0.50	1.6	0.6–3.9	0.27
Malmö	170	43	25	127	75	1.4	0.7–2.8	0.27	0.8	0.3–2.0	0.68
Gender											
Female	305	69	23	236	77	Ref					
Male	282	96	34	186	66	1.8	1.2–2.6	**0.002**			
Type of infection											
Meningitis	454	92	20	362	80	Ref					
Encephalitis	129	72	56	57	44	5.0	3.2–7.6	** < 0.001**	7.5	4.2–13.1	** < 0.001**
Unspecified CNS infection	4	1	25	3	75	1.3	0.1–12.8	1.00	3.8	0.1–149	0.47
Borrelia serology performed											
No	179	23	13	156	87	Ref					
Yes	183	104	57	79	43	8.9	5.2–15.2	** < 0.001**	9.1	4.8–17.1	** < 0.001**
No data	225	38	17	187	83	1.4	0.7–2.5	0.26	0.2	0.07–0.7	**0.005**
Time period											
2000–2004	232	40	17	192	83	Ref					
2005–2008	220	72	33	148	67	2.3	1.5–3.7	** < 0.001**	3.0	1.6–5.3	**0.001**
2009–2012	135	53	39	82	61	3.1	1.9–5.1	** < 0.001**	2.4	1.2–4.9	**0.013**
Department of pediatrics											
City											
Helsingborg	43	8	19	35	81	Ref					
Kristianstad	35	8	23	27	77	1.3	0.4–3.9	0.64			
Lund	55	7	13	48	87	0.6	0.2–2.0	0.42			
Malmö	41	5	12	36	88	0.6	0.1–2.1	0.42			
Gender											
Female	70	13	19	57	81	Ref					
Male	104	15	14	89	86	0.7	0.3–1.7	0.46			
Type of infection											
Meningitis	85	11	13	74	87	Ref					
Encephalitis	88	16	18	72	82	1.5	0.6–3.5	0.34			
Unspecified CNS infection	1	1		0		NA	NA	NA			
Borrelia serology performed											
No	50	5	10	45	90	Ref					
Yes	44	13	30	31	70	3.8	1.2–11.7	**0.016**	3.8	1.2–11.7	**0.023**
No data	80	10	12	80	88	1.3	0.4–4.1	0.66	1.3	0.4–4.1	0.66
Time period											
2000–2004	95	15	16	80	84	Ref					
2005–2008	44	6	11	38	89	0.8	0.3–2.4	0.74			
2009–2012	35	7	20	28	80	1.3	0.4–3.7	0.57			

*CI*, confidence interval; *n*, number of cases; *NA*, not applicable; *OR*, odds ratio; *TBE*, tick-borne encephalitis; Bold indicates a *p*-value <0.05

In the multivariate analysis, the independent variables associated with having had a TBE serology performed at the departments of infectious diseases were being treated at Kristianstad Hospital, diagnosed with encephalitis, having had a *Borrelia* serology performed, and being ill during 2005–2012 (Table 3). In the univariate analysis, male gender was associated with having had TBE serology performed, but did not fall out in the multivariate analysis. For the departments of pediatrics, the only independent variable associated with having had a TBE serology done was performed *Borrelia* serology at the time of illness (Table 3).

Overall, 96 out of 208 (46%) cases that had fallen ill during March to December, and who were tested with *Borrelia* serology, were *not* tested for TBE at the time of the infection.

In the sub-population of those who were tested with *Borrelia* serology at the departments of infectious diseases at the time of infection, the independent variables encephalitis and being treated at Kristianstad Hospital were associated with being tested for TBE (Table 4).

**Table 4:** Univariate and multivariate analysis with performed TBE serology as dependent variable in the sub-population at departments of infectious diseases where *Borrelia* serology was performed, indicating tick exposure

	Total	TBE serology performed		TBE serology not performed		Univariate analysis			Multivariate analysis		
		*n*	%	*n*	%	OR	95% CI	*p*-value	OR	95% CI	*p*-value
City											
Helsingborg	22	11	50	11	50	Ref					
Kristianstad	20	17	85	3	15	5.7	1.2–25.1	**0.016**	8.6	1.7–43.4	**0.009**
Lund	56	35	62	21	38	1.7	0.6–4.6	0.31	2.2	0.7–7.1	0.18
Malmö	85	41	48	44	52	0.9	0.3–2.4	0.88	1.0	0.3–3.1	0.98
Gender											
Female	79	36	46	43	54	Ref					
Male	104	68	65	36	35	2.3	1.2–4.2	**0.007**	2.0	1.0–4.0	0.05
Type of infection											
Meningitis	124	53	43	71	57	Ref					
Encephalitis	58	50	86	8	14	8.4	3.6–19.2	** < 0.001**	9.4	3.9–22.5	** < 0.001**
Unspecified CNS infection	1	1		0		NA	NA	NA			
Time period											
2000–2004	43	21	49	22	51	Ref					
2005–2008	66	34	52	32	48	1.1	0.5–2.4	0.78			
2009–2012	74	49	66	25	34	2.0	0.9–4.5	0.064			

*CI*, confidence interval; *NA*, not applicable; *n*, number of cases; *OR*, odds ratio; *TBE*, tick-borne encephalitis; Bold indicates a *p*-value < 0.05

### Subjects with retrospective TBE serology

For those where TBE serology had not been performed at the time of infection, it could be performed retrospectively in 137 out of 502 (27%) of those infected during March to December. Finally, two out of 137 retrospectively analyzed cases turned out to be both IgM and IgG positive for TBEV, indicating TBE being the actual cause of the CNS infection. One of them was a 69-year-old man already diagnosed in Germany and transferred to hospital in Skåne County during 2004 for further care. The other one was a 30-year-old woman treated for meningitis in 2005. She told not having been vaccinated against TBE, and she had neither been outside Skåne during the incubation period nor visited a previously known risk area for TBE in Skåne County. She was a dog owner and used to walk the dog in parks in the city of Malmö, and during the incubation period also nearby lake Vomb, about 35 km east of Malmö (Fig. 1B). Three cases were positive in IgG but negative in IgM, indicating a prior TBE vaccination, a passed TBE infection, or cross reactivity with other flavi viruses.

## Discussion

Only one case of undiagnosed TBE infection was found in the retrospective analysis (1/137). This low prevalence is in accordance with comparable studies in other European countries and regions in Sweden [17, 18]. If the same prevalence, hypothetically, could be applied on the 365 cases, where retrospective TBE serology was not possible to be performed, additionally, three undiagnosed TBE cases could be expected. During the same period, 2000–2012, a total of 50 cases of TBE were diagnosed in the routine health care in Skåne County. Thus, only a minority of TBE cases manifested as a CNS infection escaped detection during 2000–2012. Considering the overall low rate (25%) of TBE serologies performed among patients with CNS infections in Skåne County during the study period, this is an unexpected result. However, our results manifest TBE as a rare disease in Skåne County during the study period and support an assumption that TBEV was recently introduced in Skåne. The first risk area in Skåne County was described in 2002 in the northeastern part of the region by physicians at the department of infectious diseases in Kristianstad and there are only single reports of TBE in Skåne County before the year 2000 [12]. In a retrospective study of 29 patients with viral meningoencephalitis of unknown etiologic agent in northeastern Skåne 1997–2000, TBE was only found in one case when TBE serology was performed retrospectively [18]. That person had most probably contracted the disease when visiting Stockholm archipelago, a well-known, endemic area for TBE. This further supports that TBE was recently introduced in Skåne. However, during the following years, TBE has spread to new areas in Skåne county as shown in Fig. 1. The single case of TBE in our study that was retrospectively diagnosed had not been in any known risk area for TBE during the incubation period and demonstrates the importance of considering TBE as a differential diagnosis even outside known risk areas.

Furthermore, our data shows that although a tick exposure was considered possible since *Borrelia* serology was performed, TBE serology was only analyzed in 52% of those cases. Our study also shows that TBE was more often considered a differential diagnosis in the northeastern part of Skåne County, where the first risk area was described and where most TBE cases have occurred. This raises concerns about the vigilance on and surveillance of new tick-borne infections introduced in new areas. In Skåne County, as well as in the rest of Sweden, all patients with TBE are thoroughly interviewed to assess as close as possible the place where the infection most probably was contracted. This information is then used to assess risk areas for TBE infection and to communicate vaccination recommendations to the public. Hence, it is of great importance to diagnose all cases of TBE. This is of special importance in regions with low incidence of the disease as it gives the opportunity for early detection of previously unknown risk areas. In this respect, it is important to diagnose TBE also among those with less severe clinical manifestations. Our data suggests that TBE is less likely considered in cases of meningitis, which is a more common clinical manifestation of TBEV infection in younger ages [19, 20]. In Sweden, the peak of the TBE season overlaps the enterovirus season, and hence, TBE cases could easily be mistaken for enterovirus meningitis among children and youths if not analyzed for.

TBE is in many studies from different countries, including Sweden, described as more common among men than women [9, 17, 19–24]. Although more frequent tick exposure among males is sometime suggested as an explanation, the full explanation of this uneven sex ratio is not known [24]. In our study with cases of CNS infection of unknown etiology, we found an overall equal gender distribution among adults. However, there was a predominance of men diagnosed with encephalitis and adult women diagnosed with meningitis. The difference in gender distribution regarding the type of CNS infection with unknown etiology requires further studies. McGill et al. have in a prospective study previously observed female predominance in viral meningitis as well as in meningitis of unknown cause [4]. In our study, encephalitis, but not gender, fell out in the multivariate analysis as an independent variable associated with performed TBE serology. This raises the possibility that the skewed gender distribution among TBE cases partly could be attributed to underdiagnosing TBE in women with meningitis as the principal manifestation of TBE.

Our study has some limitations which need to be considered in the interpretation of the results. Firstly, the study population was chosen as all patients with specified ICD-10 codes, treated at the departments of infectious diseases and pediatrics in Skåne County during a specified period. Most patients with viral CNS infections can be assumed to be treated at, or referred to, these clinics. However, some cases with the specified ICD-10 codes could have been treated at departments of neurology or internal medicine, and thereby not been reached in this study. Secondly, except for the above-mentioned process of excluding patients with already identified etiological virus, we did not assess the correctness of the given ICD-10 code. However, none of the patients that were asked for informed consent to use their stored blood sample for TBE serology objected with reference to not having had a CNS infection. Finally, there is a risk of recall bias regarding possible places of infection from the patient diagnosed with TBE due to the time elapsed since possible tick exposure. The study period chosen comprises the period when the first risk area for TBE was identified and the following years when TBE was spread to new areas in Skåne. It was not possible to include even earlier years since data from the current diagnosis registration system only include data from year 2000 and onwards.

The generalizability to other geographic areas regarding number of missed TBE diagnoses has to be done with cautiousness. Regions with different incidence and endemicity of TBE present a different risk for TBE after tick exposure and the physicians probably have another awareness of the disease. However, the aim of this study was not to predict missed TBE diagnoses in other regions but to estimate the awareness of the disease in a region with emerging risk areas, and to find out whether neglected TBE diagnoses could give valuable information on assessing risk areas for TBE. Nevertheless, results from our study suggest that when new viral pathogens causing CNS infections are introduced in an area, e.g., due to climate changes, there is an obvious risk of missing its spread if not a thorough microbiological investigation is performed.

## Conclusion

In conclusion, the low number of retrospectively diagnosed cases in this study confirms TBE as a disease with low incidence at the study period and indicates that TBEV probably was recently introduced in Skåne County. Our data show an insufficient TBE sampling among patients with presumed viral CNS infection of unknown etiology, especially in cases with suspected tick exposure. A higher awareness of TBE as a differential diagnosis could contribute to earlier detection of new risk areas and adequate preventive advice to the public, including TBE vaccine recommendations. We suggest that, if there is any possibility of tick exposure and no other etiologic agent is found, all patients with presumed viral CNS infections are tested for TBE.

## Data Availability

The datasets generated during and/or analyzed during the current study are available from the corresponding author on reasonable request.
